# Bilateral renal mucormycosis following COVID-19 infection: A therapeutic challenge 

**DOI:** 10.5414/CNCS110874

**Published:** 2022-11-24

**Authors:** Rajasekaran Kishor Kumar, Rajeev A. Annigeri, Ram Gopalakrishnan, Sunil S. Kaveripattu, Nitesh Jain

**Affiliations:** Departments of; 1 Nephrology,; 2 Infectious diseases,; 3 Histopathology, and; 4 Urology, Apollo Hospitals, Chennai, India

**Keywords:** COVID-19, renal mucormycosis, amphotericin

## Abstract

India witnessed an epidemic of mucormycosis during the second wave of the COVID-19 pandemic. Renal mucormycosis has been reported rarely, mostly from India, but only 2 cases have been reported following COVID-19 infection to date. We report a case of mucormycosis predominantly affecting kidneys in a young and previously healthy male following COVID-19 pneumonia, for which he had received corticosteroid, remdesivir, and tocilizumab. He presented with hematuria, progressive oliguria, and severe acute kidney injury (AKI) requiring dialysis. The diagnosis was made on kidney biopsy and contrast-enhanced CT (CECT) showed segmental and subsegmental renal artery pseudoaneurysms with distal occlusion of both kidneys. He underwent bilateral nephrectomy and received high-dose amphotericin (AMB) and posaconazole. He developed cardiac arrhythmia and pulmonary edema attributed to AMB-related cardiotoxicity after a cumulative ABM dose of 2,450 mg. This is the first case report describing the survival of a patient with bilateral renal mucormycosis following COVID-19 infection. Our case report highlights the importance of considering mucormycosis in a patient with post-COVID-19 AKI to make an early diagnosis and aggressive management comprising of surgical debridement and high-dose AMB to improve survival.

## Introduction 

Mucormycosis is a rare, but potentially life-threatening fungal infection, more often encountered in immunocompromised patients such as organ transplant recipients and patients with uncontrolled diabetes mellitus. With the ongoing COVID-19 pandemic, India witnessed an unprecedented epidemic of mucormycosis in patients who recovered from COVID-19 infection, during the second wave due to Delta variant in 2021 [1]. 

Renal involvement in mucormycosis is very rare and is a unique feature of mucormycosis in India, which accounts for the vast majority of such cases [2, 3]. Post-COVID renal mucormycosis is extremely rare, and only 2 cases have been reported to date [4, 5]. We describe a case of a young adult male who developed bilateral renal mucormycosis following COVID-19 pneumonia, and we discuss the management strategy, which resulted in the survival of the patient. 

## Case report 

A 31-year-old male with no known comorbidities was admitted to another hospital in the summer of 2021 with fever for 5 days and breathlessness for 2 days. He was diagnosed to have COVID-19 pneumonia based on the positive nasopharyngeal swab for COVID-19 RT-PCR. Initial CT showed bilateral alveolar ground glassing and infiltrates involving 28% of lung, without any nodules. The patient received a 200-mg loading dose remdesivir followed by 100 mg daily for the next 4 days and oral dexamethasone 8 mg twice daily for 10 days, a dose double that recommended by standard guidelines. He had progressive dyspnea, requiring non-invasive ventilation with FiO_2_ of 60%. IL-6 level was high (16.5 pg/mL, normal: < 5.9), and he received a single dose of intravenous tocilizumab 400 mg (5.3 mg/kg). He was discharged after 21 days, at which time serum creatinine (SCr) was 0.8 mg/dL. Two days after discharge, he developed gross hematuria and progressive oliguria over the next 3 days, and SCr was 10.8 mg/dL. He was hospitalized and was initiated on hemodialysis through internal jugular catheter. Ultrasound study showed normal-sized kidneys with increased echoes and a normal-to-low resistive flow pattern in the intrarenal arteries. A renal biopsy was done, which showed infarction involving 35% of cortex, and aseptate fungal hyphae which showed right-angle branching and irregular outlines embedded in the necrosed tissue suggestive of mucormycosis. Urine culture on Sabouraud’s dextrose agar media grew *Mucor* species. He received intravenous conventional amphotericin-B (AMB) at 50 mg initially which was increased to 100 mg/day after 5 days. He subsequently was transferred to our hospital for further management. 

**Figure 1.**
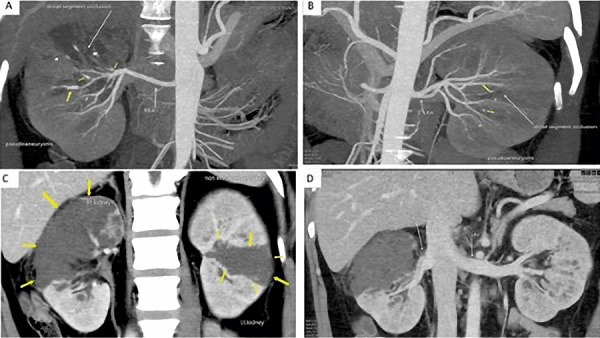
Contrast CT abdomen showing bilateral small pseudoaneurysms in the segmental branches of the renal arteries (A, B) with bilateral renal infarcts (C) with patent bilateral renal vein (D).

**Figure 2.**
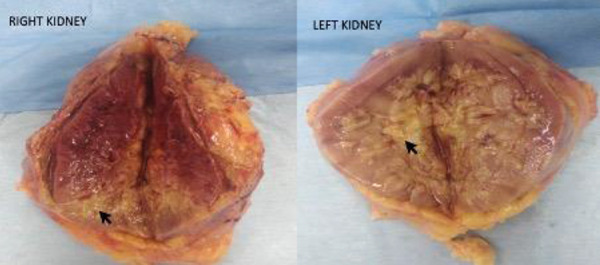
Cut section of the nephrectomy specimen showing loss of corticomedullary differentiation, replacement of normal parenchyma by necrosed tissue (arrow).

**Figure 3.**
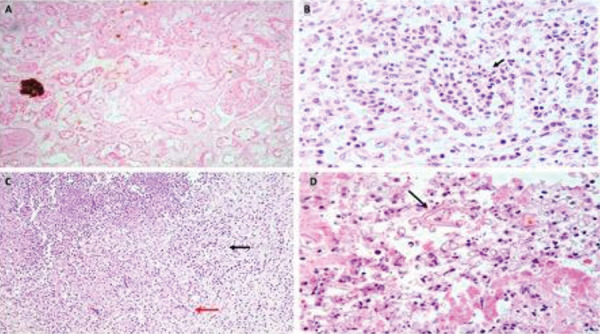
A: H & E stain, × 100, microphotograph showing patchy areas of extensive cortical necrosis with intratubular neutrophilic aggregates. B: H & E stain, × 100, microphotograph showing focal area of intratubular neutrophilic aggregates (black arrow) with tubular rupture suggestive of acute pyelonephritis. C: H & E stain, × 100 microphotograph showing suppuration with surrounding epithelioid histiocytic aggregates (black arrow) and multinucleated giant cells (red arrow). D: H & E stain, × 100, microphotograph showing focus of angio-invasive broad aseptate fragmented, irregular branching fungal hyphal elements (black arrow) with morphology consistent with *Mucor* species.

At admission, blood pressure was 128/84 mmHg, pulse rate was 102/minute, respiratory rate was 22/minute, oxygen saturation at room air was 96%, and he weighed 79.3 kg. Physical examination showed pallor, mild pedal edema, and few basilar rales over the chest on auscultation. Investigations showed blood urea 158 mg/dL, SCr 6.5 mg/dL, serum sodium 130 meq/L, serum potassium 5.4 meq/L, serum bilirubin 0.5 mg/dL, serum ALT 60 U/L, serum procalcitonin 1.27 ng/mL, serum ferritin 1,610 ng/mL, hemoglobin 9.3 gm/dL, and WBC count 38,200/mm^3^ (N 82%, L 3%, M 8%, B 1%, myelocytes 4%, metamyelocytes 2%). The echocardiography showed a normal left ventricular (LV) size and ejection fraction (EF). He was oliguric and dialysis dependent. The contrast-enhanced CT (CECT) angiography showed bilateral renal infarcts with multiple small pseudoaneurysms in bilateral segmental and subsegmental renal arterial branches with distal occlusion and no evidence of renal vein thrombosis (Figure 1). CT chest and pulmonary angiography showed diffuse ground-glass opacities with fibrotic strands and tractional bronchiectasis suggestive of sequelae to COVID-19 pneumonia. His health showed rapid deterioration, and 3 days later laparoscopic bilateral nephrectomy was done for source reduction of mucormycosis, following which he improved. Gross examination of the nephrectomy cut specimen showed loss of corticomedullary distinction and renal was replaced by necrosis and yellowish-black areas involving parts of the kidneys (Figure 2). Histology of the nephrectomy specimen showed cortical infarction and fungal hyphae consistent with mucormycosis (Figure 3). He continued to receive conventional AMB 100 mg (1.5 mg/kg) infusion over 4 hours daily which was later modified to 150 mg 3 times a week given after dialysis. Posaconazole delayed-release capsule 300 mg/day was added to the antifungal regimen. He developed an abscess over the right leg, and the aspirate showed necrotic tissue with pigmented broad aseptate fungal hyphae. He developed acute-onset breathlessness and an episode of syncope and bradyarrhythmia 45 days after initiation of AMB and a cumulative dose of 2,450 mg. Echocardiography showed severe LV dysfunction with an EF of 45% and a wide variation in heart rate. Serum potassium was 3.9 meq/L, and serum pro-BNP was > 35,000 pg/mL (normal: < 125). Initially, the fluid overload was considered as the cause of pulmonary edema, and aggressive fluid removal during dialysis was done. AMB was stopped since he was subsequently considered to have AMB-related cardiomyopathy since he had persistent dyspnea and cardiac arrhythmias which required hospitalization. One month after the withdrawal of AMB, he had a normal LV function. He remained well 6 months after the initial COVID-19 infection while receiving hemodialysis and posaconazole. 

## Discussion 

### COVID-19-associated mucormycosis in India 

India witnessed an epidemic of mucormycosis during the COVID-19 pandemic, due to the delta variant in 2021. This was attributed to poorly controlled diabetes mellitus, overuse of corticosteroids and other immunosuppressants, iron overload, and environmental factors such as unsanitary conditions [1]. Muthu et al. [6] reviewed 275 cases of COVID-19-associated mucormycosis (CAM) published in the literature until June 2021, of which 85% were from India. The major presentation of CAM in India was rhino-orbital cerebral mucormycosis in 89%, followed by pulmonary in 7.3% and 4.3% in other organs [6]. 

### Renal mucormycosis in COVID-19 infection 

The precise incidence of mucormycosis in India is not known but is higher than in other countries [1]. Most cases of renal mucormycosis reported in the literature are from India and the reported mortality is high at 50 – 60% [1]. Though isolated mucormycosis has been reported in non-COVID-19 patients predominantly from India, only 2 cases of renal mucormycosis following COVID-19 infection have been reported previously. Both of these patients received corticosteroid of unspecified dose and none received tocilizumab. Singh et al. [4] reported a case of a 32-year-old male who presented with acute abdomen 4 weeks after COVID-19 infection. CECT showed infarction and renal artery thrombosis of the right kidney. He underwent a right nephrectomy but expired soon after, and the microscopic examination of the renal tissue revealed mucormycosis. Choudhary et al. [5] reported a case of a 32-year-old man who presented with low-grade fever and flank pain 45 days after COVID-19 infection. Ultrasonography showed enlarged left kidney and absent flow in the left renal vein on Doppler study, for which he received intravenous heparin. CT scan showed features of acute pyelonephritis of the left kidney, and a ureteric stent was inserted. He continued to deteriorate and underwent a left nephrectomy that revealed renal mucormycosis. His condition deteriorated post surgery leading to death. 

Our patient was a young male and received twice the recommended dose of corticosteroids for 10 days and additionally tocilizumab, which is reported to increase the risk of fungal infections after COVID-19 infection [7, 8]. He presented with hematuria and progressive renal failure requiring dialysis. The early diagnosis was made by kidney biopsy, and CECT showed renal artery occlusions, aneurysms, and infarctions in both kidneys. In addition to kidney involvement, there was evidence of hematogenous disseminated disease in the form of cutaneous lesions. We hypothesize that the excessive dose of corticosteroids, especially in combination with IL-6 inhibitors like tocilizumab might have contributed to the development of mucormycosis. This reinforces the importance of following guideline-recommended doses of immunomodulatory agents in COVID-19 to avoid superadded infections. 

### Management of renal mucormycosis and therapy-related complications 

The management of mucormycosis involves early initiation of therapy, surgical debridement of infected tissue, anti-fungal medications, and management of predisposing medical conditions such as diabetes mellitus and immunosuppression. AMB is the mainstay of treatment for invasive mucormycosis [9]. The best outcomes are seen in patients who received combined AMB and surgical debridement [1]. We adopted an aggressive approach of combined bilateral nephrectomy to limit the source of infection and high-dose AMB combined with oral posaconazole, which resulted in a successful outcome. Our case is the first case of post-COVID-19 renal mucormycosis with a successful outcome and highlights the importance of an early diagnosis and aggressive approach of combined surgical debridement and high-dose AMB. 

AMB is known to produce cardiac arrhythmias, especially following a rapid infusion in patients with kidney failure [10]. Six cases of reversible cardiomyopathy have been reported in the literature and were reviewed by Bandeira recently [11]. The time duration of AMB treatment to cardiomyopathy varied from 7 days to 8 months. The pathogenic mechanisms of AMB-induced cardiomyopathy are not clear, and it is speculated that the interaction of AMB with cholesterol-containing cell membranes may result in cellular injury and end-organ dysfunction [12]. Our patient developed cardiomyopathy after a cumulative dose of 2,450 mg of AMB, which reversed within 4 weeks after stopping AMB. Our case report highlights the risk of cardiac toxicity, when AMB is given in high doses in anuric patients. The options for patients with AMB intolerance or failures are the azoles, isavuconazole and posaconazole [13]. Though not recommended as combination therapy or as initial sole therapy, these are useful agents for long-term treatment; our patient was successfully treated with posaconazole after AMB was discontinued. 

## Conclusion 

We report a case of renal mucormycosis following COVID-19 pneumonia in a young previously healthy male, who was successfully treated with bilateral nephrectomy and high-dose AMB and posaconazole but developed reversible cardiomyopathy necessitating discontinuation of AMB. Our case report highlight the importance of considering mucormycosis in a patient with post-COVID-19 AKI to make an early diagnosis and aggressive management of surgical debridement and high-dose AMB which could improve the survival in this potentially life-threatening condition. 

## Acknowledgment 

We would like to express our gratitude to Dr. Geetha Narasimhan, pathologist, for her contribution to the histological reporting of the case. 

## Funding 

The case report was not funded by any individual, organization or agency. 

## Conflict of interest 

None of the authors have any conflict of interest in the preparation and publication of this case report.
